# *SNF3* as High Affinity Glucose Sensor and Its Function in Supporting the Viability of *Candida glabrata* under Glucose-Limited Environment

**DOI:** 10.3389/fmicb.2015.01334

**Published:** 2015-12-01

**Authors:** Tzu Shan Ng, Shu Yih Chew, Premmala Rangasamy, Mohd N. Mohd Desa, Doblin Sandai, Pei Pei Chong, Leslie Thian Lung Than

**Affiliations:** ^1^Department of Medical Microbiology and Parasitology, Faculty of Medicine and Health Sciences, Universiti Putra MalaysiaSerdang, Malaysia; ^2^Department of Biomedical Sciences, Faculty of Medicine and Health Sciences, Universiti Putra MalaysiaSerdang, Malaysia; ^3^Infectomics Cluster, Advanced Medical and Dental Institute, Universiti Sains MalaysiaBertam, Malaysia

**Keywords:** *Candida glabrata*, glucose sensor, *SNF3*, glucose-limited environment, hexose transporter

## Abstract

*Candida glabrata* is an emerging human fungal pathogen that has efficacious nutrient sensing and responsiveness ability. It can be seen through its ability to thrive in diverse range of nutrient limited-human anatomical sites. Therefore, nutrient sensing particularly glucose sensing is thought to be crucial in contributing to the development and fitness of the pathogen. This study aimed to elucidate the role of *SNF3* (Sucrose Non Fermenting 3) as a glucose sensor and its possible role in contributing to the fitness and survivability of *C. glabrata* in glucose-limited environment. The *SNF3* knockout strain was constructed and subjected to different glucose concentrations to evaluate its growth, biofilm formation, amphotericin B susceptibility, *ex vivo* survivability and effects on the transcriptional profiling of the sugar receptor repressor (SRR) pathway-related genes. The *CgSNF3*Δ strain showed a retarded growth in low glucose environments (0.01 and 0.1%) in both fermentation and respiration-preferred conditions but grew well in high glucose concentration environments (1 and 2%). It was also found to be more susceptible to amphotericin B in low glucose environment (0.1%) and macrophage engulfment but showed no difference in the biofilm formation capability. The deletion of *SNF3* also resulted in the down-regulation of about half of hexose transporters genes (four out of nine). Overall, the deletion of *SNF3* causes significant reduction in the ability of *C. glabrata* to sense limited surrounding glucose and consequently disrupts its competency to transport and perform the uptake of this critical nutrient. This study highlighted the role of *SNF3* as a high affinity glucose sensor and its role in aiding the survivability of *C. glabrata* particularly in glucose limited environment.

## Introduction

Glucose is commonly known as an important carbon source and energy for many organisms. Several studies have attempted to establish the linkage between glucose availability and physiological response of *Candida* species, including the biofilm formation, oxidative stress, and antifungal resistance (Rodaki et al., 2009; Uppuluri et al., 2010; Ene et al., 2012; Ng et al., 2015b). The regulatory effect by glucose found in these studies is suggestive of the importance of glucose sensing and uptake mechanism in contributing to the fitness of *Candida* species. Brown et al. (2006) have demonstrated that the loss of Hgt4, a high affinity glucose sensor resulted in a less virulent *C. albicans* type that failed to grow in low glucose and fermentation-preferred environments. In addition, the loss of Hgt4 also affects the ability of *C. albicans* to perform the yeast-hyphal morphological switch and therefore compromises its pathogenicity in mouse model of disseminated candidiasis (Brown et al., 2006). Apart from that, the ability to transport glucose by *Cryptococcus neoformans* is also diminished with the loss of Hxs1, a high affinity glucose sensor-like protein (Liu et al., 2013). The diminished glucose uptake activity leads to an attenuated strain of *C. neoformans* in which the strain demonstrated a delay in lethal infection in mice model. However, little is known about the role of high affinity glucose sensor in the emerging human fungal pathogen, *Candida glabrata*.

The ability of *C. glabrata* to thrive in several glucose-limited anatomical sites of host such as vaginal and blood (Ehrström et al., 2006) is suggestive of its sensitivity toward the low glucose availability in the niches with its superior glucose sensing ability. The primary mechanism for *Saccharomyces cerevisiae* to sense and transport surrounding glucose is through *SNF3*-*RGT2* mediated sugar receptor repressor (SRR) pathway (Rolland et al., 2002; Santangelo, 2006; Gancedo, 2008). This pathway employs two glucose sensors with different affinity toward glucose: *SNF3* (high affinity) and *RGT2* (low affinity). They are located in the cell membrane and modulate the expression of the hexose transporters (*HXT*s) for the uptake of glucose through the interplay of transcription regulators (*RGT1* and *MIG1*) and downstream component of SRR: *YCK1* and *YCK2* (casein kinase), *GRR1* (Glucose Repression Resistant), *STD1* (repressor of *RGT1*; summarized and illustrated in **Figure 9**; Schmidt et al., 1999; Kim and Johnston, 2006). The homologs of these key genes were found in the *C. glabrata* genome. The phylogenetic analysis conducted demonstrates the shared neighborhood between Cg*SNF3* (sequence ID: CAGL0J09020g) and Sc*SNF3* (Palma et al., 2009; Ng et al., 2015a). In addition, the key feature of glucose sensor as found in Sc*SNF3*, for example the unusual long C-terminal segment amino acids and the signature Özcan motif were also found in the Cg*SNF3* (Palma et al., 2009; Ng et al., 2015a). Nevertheless, these studies have only managed to highlight the phylogenetic relatedness between Sc*SNF3* and Cg*SNF3*, but the actual physiological role of *SNF3* in *C. glabrata* is remains unknown. In respect to the importance of glucose in cell physiology, the disruption in SRR pathway may have negative impact on the fitness of *C. glabrata*. Therefore, this study aimed to explore the possible role of *SNF3* in supporting the growth and fitness of *C. glabrata* under low glucose concentration environment. Its growth profile, biofilm formation, antifungal susceptibility, and capability to withstand phagocytosis of macrophage were assessed. In addition, its role in regulating the expression of the SRR pathway-related genes, which includes the hexose transporters (*HXT*s), was also deciphered.

## Materials and methods

### Yeast strain and media preparation

*C. glabrata* BG14 (gift from Brendan Cormack, John Hopkins University; Cormack and Falkow, 1999) and its parental strain *C. glabrata* BG2 (gift from Paul Fidel, Louisiana State University Health Sciences Center) were used in this study (Table 1). Three types of media were utilized: standard YPD (Becton, Dickinson and Company, USA; 20 g of peptone, 20 g of glucose, 10 g of yeast extract), synthetic minimal glucose medium, SD [0.67% of yeast nitrogen base (Becton, Dickinson and Company, USA) + glucose (Fisher Scientific, USA)] and synthetic complete media with uridine dropout [0.17% yeast nitrogen base without ammonium sulfate and amino acid (Becton, Dickinson and Company, USA) + 0.5% ammonium sulfate (Sigma Aldrich, USA) + 2% glucose (Fisher Scientific, USA) + complete supplement mixture with uridine dropout (ForMedium, UK)] (Sherman, 2002). All strains were maintained at 37°C in YPD, unless otherwise indicated.

**Table 1:** *****Candida glabrata*** strains used in this study**.

***C. glabrata***	**Genotype**	**References**
**strains**		
BG2	Wild type	Cormack and Falkow, 1999
BG14	Ura3Δ (−85+932):: Tn903NeoR	Cormack and Falkow, 1999
*SNF3Δ_a*		
*SNF3Δ_b*	Derived from BG14, *SNF3*::*URA3*	This study
*SNF3Δ_c*		

### Strain construction

For the construction of the *C. glabrata SNF3*Δ strain, the *C. glabrata* BG14 was transformed to Ura3 + by replacing the *SNF3* open reading frame (ORF) with *SNF3*::*URA3* disruption cassette. *SNF3*::*URA3* disruption cassette that consists of “upstream *SNF3*-*URA3*-downstream *SNF3*” was amplified by PCR (primers TS_SNF3_F and TS_SNF3_R; Table 2) and purified through Expin™ Combo GP purification kit (GeneAll, Korea). The purified cassette was then transformed into *C. glabrata* BG14 as described in Cormack and Falkow (1999). Transformants were selected on synthetic complete media with uridine dropout and insertion was confirmed by diagnostic PCR (primers CHK_F_1 CHK_R_1 and CHK_F_2 CHK_R_2; Table 2) for the absence of *SNF3* and presence of *URA3* at the correct locus. In order to eliminate the possible effect from secondary mutation of mutant constructed, three independently constructed *SNF3*Δ mutants were analyzed, and treated as three biological replicates in the subsequent assays (Odds et al., 2006).

**Table 2:** **List of primers used in this study**.

**Target gene name**	**Direction**	**Sequence 5′–3′**	**Expected amplification size**
*RGT2* (Restores Glucose Transport 2)	Forward	CGTTTGTGGGACTTTTCGTT	203 bp
(CAGL0I03872)	Reverse	TGAAATCCATGGAGCAATGA	
GRR1 (Glucose Repression-Resistance 1)	Forward	TTGTCGAGCTTACAGGCAGA	149 bp
(CAGL0M09130)	Reverse	CCCTCCAATCTTTGGTTTCC	
STD1 (Suppressor of Tbp Deletion 1)	Forward	GAGTGCCCCACCAGAATATG	146 bp
(CAGL0L10043)	Reverse	AGGACTGCGAGCTGTGACTT	
YCK1 (Yeast Casein Kinase 1)	Forward	CGCTGACAATGCTAACCAGA	150 bp
(CAGL0G06138)	Reverse	TGAACACGTTGTCCTTGCAT	
YCK2 (Yeast Casein Kinase 1)	Forward	TCGGAGAGACTATGGACGGTA	149 bp
(CAGL0J05940)	Reverse	GGCAGCGTTTCTGTTCCTAT	
RGT1 (Restores Glucose Transport 1)	Forward	CCAACTCAAAGGATGGAGGA	194 bp
(CAGL0L01903)	Reverse	TATCGTTGGCGTCATTTTGA	
MIG1 (Multicopy Inhibitor of GAL gene expression 1)	Forward	CCGGGATGTGTCAAGAGATT	212 bp
(CAGL0A01628)	Reverse	CGTTTCGTCTTCCTCCTCAG	
*HXT1* (Hexose Transporter 1)	Forward	AAACCAAGTCGGCAAGAATG	224 bp
(CAGL0A01804)	Reverse	ATTCAGTTCCGTCAGGATGC	
*HXT3* (Hexose Transporter 3)	Forward	TGACCTTCGTTCCAGAATCC	165 bp
(CAGL0A0231)	Reverse	TACCAGCGGCATTAGCTTCT	
*HXT5* (Hexose Transporter 5)	Forward	TATGTTTCGCATGGGCATTA	153 bp
(CAGL0A01826)	Reverse	CCAAAAGGACGATTGGAGAA	
*HXT4* (Hexose Transporter 4)	Forward	TCCTGGGGTGAATTGTTCTC	228 bp
(CAGL0A01782)	Reverse	GCCAAATCTACCGACCAAGA	
HXT6/7 (Hexose Transporter 6/7)	Forward	GCTTCGGTCGTCGTAAATGT	195 bp
(CAGL0A00737/A02233)	Reverse	GAGTTGGTGCCCAAGTTGTT	
HXT6/7 (Hexose Transporter 6/7)	Forward	GGTCAAGACCAACCATCCTCC	182 bp
(CAGL0A02211)	Reverse	CCCCAGATCCAGTTGGAAGC	
HXT2/10 (Hexose Transporter 2/10)	Forward	AAGCTGGAAGGCGAAGATTT	146 bp
(CAGL0I00286)	Reverse	TCCCAACCAAAGACAAAACC	
HXT2/10 (Hexose Transporter 2/10)	Forward	TGCCGAAACCTACCCACTAC	147 bp
(CAGL0D02662/D02640)	Reverse	CAGCCCATGAAGACGTAACC	
HXT14 (Hexose Transporter 14)	Forward	TACGCCAGCACACTAAAGCA	153 bp
(CAGL0M04103)	Reverse	TTGCAGAGGACACAATCGTC	
UBC13 (Ubiquitin-Conjugating 13) (CAGL0G08063)	Forward	TGCCCGAGGACTACCCTATG	100 bp
	Reverse	AGCACGTCCAGGCAGATACG	
ACT1 (ACTin 1)	Forward	TTGCCACACGCTATTTTGAG	225 bp
	Reverse	ACCATCTGGCAATTCGTAGG	
TS_SNF3	Forward	CATGGCTGGAACTAGGCGCTTATTGACGGGTATTGGAGACTTAGGATAGAGGAAGATTTTGGCATAGGATGTCCAGTGCCTCATATTTAC	–
	Reverse	GCTGCGTCTGATCGTTGTCGTTTTGTGAGTACCCTGTATTTTGGCTGGTATAGGTATTACTCTTCAGTTTCCTATTCTTTTCAAGTAAGC	–
CHK_F_1	Forward	AGCAGAGGACTCCCTCAATG	–
CHK_R_1	Reverse	TTTCAGCAACTTGGAAGCAA	–
CHK_F_2	Forward	GATACAGGAACAACAGCGAG	–
CHK_R_2	Reverse	CCATGAGCGTTGGTGATATC	–

### Growth profiling and growth rate calculation

The capability of *SNF3*Δ strain and parental wild type BG2 to grow in different levels of surrounding glucose was assessed using a modified procedure as described in Brown et al. (2006) and Rodaki et al. (2009). *C. glabrata* cells were grown overnight in YPD medium at 37°C and washed with phosphate buffered saline (PBS, pH 7.4) for three times before re-inoculated into 50 mL fresh SD (OD_600_ = 0.1) with prepared glucose concentrations of 0.01 (extremely low), 0.1 (low), 0.2 (moderate), 1 (high), and 2% (extremely high), respectively. The cells were allowed to grow at 37°C, 200 rpm in shaking incubator (shaking). Another set of cells were incubated in the same manner but were left to be static. These two conditions served as respiration-preferred (shaking) and fermentation-preferred (static) atmosphere, respectively (Brown et al., 2006). The cells were harvested hourly for 10 h and the optical density of cells (OD_600_) for each hour were recorded and proceeded with the growth rate calculation as shown in Equation (1) (Widdel, 2010).

(1) $$\begin{array}{r} \mu =\frac{2.303\;(\mathrm{lg}{\text{OD}}_{2}-\mathrm{lg}{\text{OD}}_{1})}{{\text{t}}_{2}-{\text{t}}_{1}} \end{array}$$

where,

μ = growth rate, h^−1^

OD_1_ = optical density obtained from the log phase of growth curve

OD_2_ = two times of OD_1_ obtained from the log phase of growth curve

t_1_ = time of OD_1_ obtained

t_2_ = time of OD_2_ obtained.

### Biofilm formation

The biofilm formation assay were performed with minor modification as described in Pierce et al. (2008) by replacing RPMI1640 medium with SD (0.01 and 0.1% glucose). Overnight grown *C. glabrata* cells were harvested and washed prior to re-inoculation into the defined SD (OD_600_ = 0.1). A volume of 100 μl of cell suspension was added to microtitre plate (U-shaped, tissue culture treated) (Becton, Dickinson and Company, USA). The microtitre plate was covered with lid and sealed with parafilm, followed by incubation for 24 h at 37°C. The media was aspirated and the plate was washed three times using 200 μl of PBS, pH 7.4. The plate was placed in an inverted position on a blotting paper to remove residual PBS. The biofilm activity was quantified via XTT 2,3-Bis (2-methoxy-4-nitro-5-sulfophenyl)-2H-tetrazolium-5-carboxanilide reduction assay. A freshly prepared 100 μL mixture of 0.5 g/L XTT (Sigma Aldrich, USA) and 10 mM menadione (Sigma Aldrich, USA) (10000:1, v/v) was added to the washed-biofilm in the microtitre plate. The plate was wrapped with aluminum foil and incubated in dark at 37°C for 3 h. A volume of 80 μL of the supernatant was transferred to a new microtitre plate and the plate was read by using microtitre plate reader (Bio-Tek, USA) at wavelength of 490 nm.

### Amphotericin B susceptibility assay

The inhibitory concentration of *C. glabrata* BG2 against amphotericin B was determined using the method as described in NCCLS (CLSI) M27-A2 by replacing the RPMI1460 with 0.1% glucose SD. The inhibitory concentration obtained was applied in the modified method from Rodaki et al. (2009) to elucidate the possible role of *SNF3* in contributing to the anti-amphotericin B susceptibility. Briefly, overnight grown *C. glabrata* cells were harvested and washed prior to re-inoculation into the 0.1% glucose SD (OD_600_ = 0.1) and regrown to OD_600_ = 0.5. Cell suspension was added to 1.5 mL centrifuge tube (Axygen, USA) and microtitre plate (U-shaped, tissue culture-treated) (Becton, Dickinson and Company, USA) together with defined concentration of amphotericin B. The centrifuge tube and microtitre plate was covered and sealed with parafilm, followed by incubation for 24 h at 37°C. CFU (colony-forming unit) was determined from cell suspension in centrifuge tube for the calculation of survival percentage. In addition, the plate was read by using microtitre plate reader at wavelength of 600 nm to examine the cell density for the confirmation of *C. glabrata* viability. The survivability percentage of *C. glabrata* was calculated by applying the formula as below:

(2) $$\begin{array}{r} \text{Survival percentage}=\frac{\text{CFU of stressed sample}}{\text{CFU of unstressed control}}\times 100\% \end{array}$$

### *Candida*-macrophage co-culture assay

The capability of both *C. glabrata* strains to withstand engulfment of macrophage was analyzed as described by Kaur et al. (2006) and Collette et al. (2014) with minor modification. Murine macrophage cells, RAW264.7 (gift from Daud Ahmad Israf Ali, Universiti Putra Malaysia) were maintained and incubated in Dulbecco's Modified Eagle's Medium (DMEM; Life Technologies, USA), supplemented with 10% Fetal Bovine Serum (FBS; Life Technologies, USA) at 37°C/5% CO_2_. Prior to the co-culture step, 5 × 10^5^ of RAW264.7 cells were seeded into 6-well plate (Becton, Dickinson and Company, USA) for 24 h at 37°C/5% CO_2_. After incubation, the washed cell was counted for the determination of cells number. For the preparation of *C. glabrata* cells, overnight grown *C. glabrata* cells were washed and regrown in fresh YPD (OD_600_ = 0.5). Harvested mid-log phase cells were washed and re-inoculated in fresh DMEM + 10% FBS for desired cell density to match the ratio of 1:1 (effector: target) prior to the co-culturing with RAW264.7 cells prepared. The mixed culture of *C. glabrata* and RAW264.7 was incubated at 37°C/5% CO_2_. In order to measure the growth of macrophage engulfed-yeast, non-engulfed yeast cell were washed away with DMEM after 2 h of incubation. The lysates of infected macrophages were scrapped and collected from wells at two time points (2 and 24 h) in ice-cold deionized water and plated on YPD agar. CFUs were determined after incubation of 24 h at 37°C and the growth ratio of engulfed cells were determined by applying the formula below:

(3) $$\begin{array}{r} \text{Growth ratio}=\frac{\text{CFU of }24\text{ h sample}}{\text{CFU of }2\text{ h control}}\times 100\% \end{array}$$

### RNA extraction

Overnight-cultured *C. glabrata* cells in YPD medium were washed and regrown in fresh YPD (OD_600_ = 0.1) to mid-log phase (OD_600_ = 0.5). The mid-log phase cells were collected, washed and re-suspended in SD (0.01% glucose) and allowed to grow at 37°C for 2 h. The collected cell was washed and RNA extraction was performed based on the described protocol in Yeast Current Protocols in Molecular Biology (Collart and Oliviero, 1993). Verification of the RNA integrity and quality were performed by visualization on 1% Tris-acetate-EDTA gel and NanoPhotometer® (Implen, Germany). RNAs were treated with Maxima H minus first strand cDNA synthesis kit with dsDNase (Thermo Scientific, USA) as described in kit manual with minor modification where RNAs were reverse transcribed with the mixture of Oligo(dT) 18 and random hexamer for the generation of full length transcripts (Resuehr and Spiess, 2003). The RNAs were also reverse transcribed with reaction suspension lacking reverse transcriptase (Non Reverse Transcriptase, NRT) and without RNA template (Non Template Control, NTC) as controls, respectively. The RNA isolation and subsequent cDNA synthesis were performed in three biological independent experiments.

### Quantitative real-time PCR (qRT-PCR)

Two reference genes were employed as internal controls namely the *ACT1* and *UBC13* (Li et al., 2012) for a more reliable and accurate normalization output. All PCR primers (Table 2) were designed to amplify target genes based on the gene sequences sourced from the *Candida* Genome Database (http://www.candidagenome.org/). The PCR efficiency using each set of primers of the respective genes was determined in two independent experiments by running a series of five-fold dilution of *C. glabrata* DNA in MiniOpticon™ Real time PCR (Bio-Rad, USA) machine. The amplification efficiency for each respective gene was determined to be between 90 and 110%. For the expression analysis of the genes, all samples were performed in technical triplicate. The total volume of each reaction was 20 μl where it contained the cDNA template, 500–600 nM primers, 2X SensiFAST SYBR No-ROX (SYBR green) master mix (Bioline, UK) and type-1 ultrapure water (Milipore, USA). The reagents mixture was placed in low-profile white strip tube (Life Technologies, USA) and allowed to amplify in two-step cycling PCR amplification (polymerase activation: 95°C for 2 min, 40 cycles of denaturation: 95°C for 5 s and annealing/extension: 60°C for 30 s). Melting curve analysis was performed to ensure no non-specific PCR products were generated. A NRT and NTC were included for each gene during the qRT-PCR analysis. For post-experimental expression analysis, normalized expression ratios were calculated based on the mathematical equation developed by Pfaffl (2001). Wild type BG2 was chosen as the reference strain (baseline) when interpreting the result for the transcript profiling. Normalized expression ratio calculated was presented in logarithms based (log_10_).

### Statistical analysis

Statistical analyses were performed using SPSS Statistics (Version 17.0) software. All the experiments were performed at least three times and the data presented are mean of all experiments performed. Error bars represent standard error of the mean (SEM). Statistical significance was assessed by unpaired *t*-test to compare control (wild type) and sample (mutant). The relative expression software tool (REST©) version 2009 (Pfaffl et al., 2002) was employed to test the statistical significance in qRT-PCR analysis.

## Results

### Loss of *SNF3* resulted in the failure of *C. glabrata* to thrive in low glucose concentration environments

**Figure 1:**
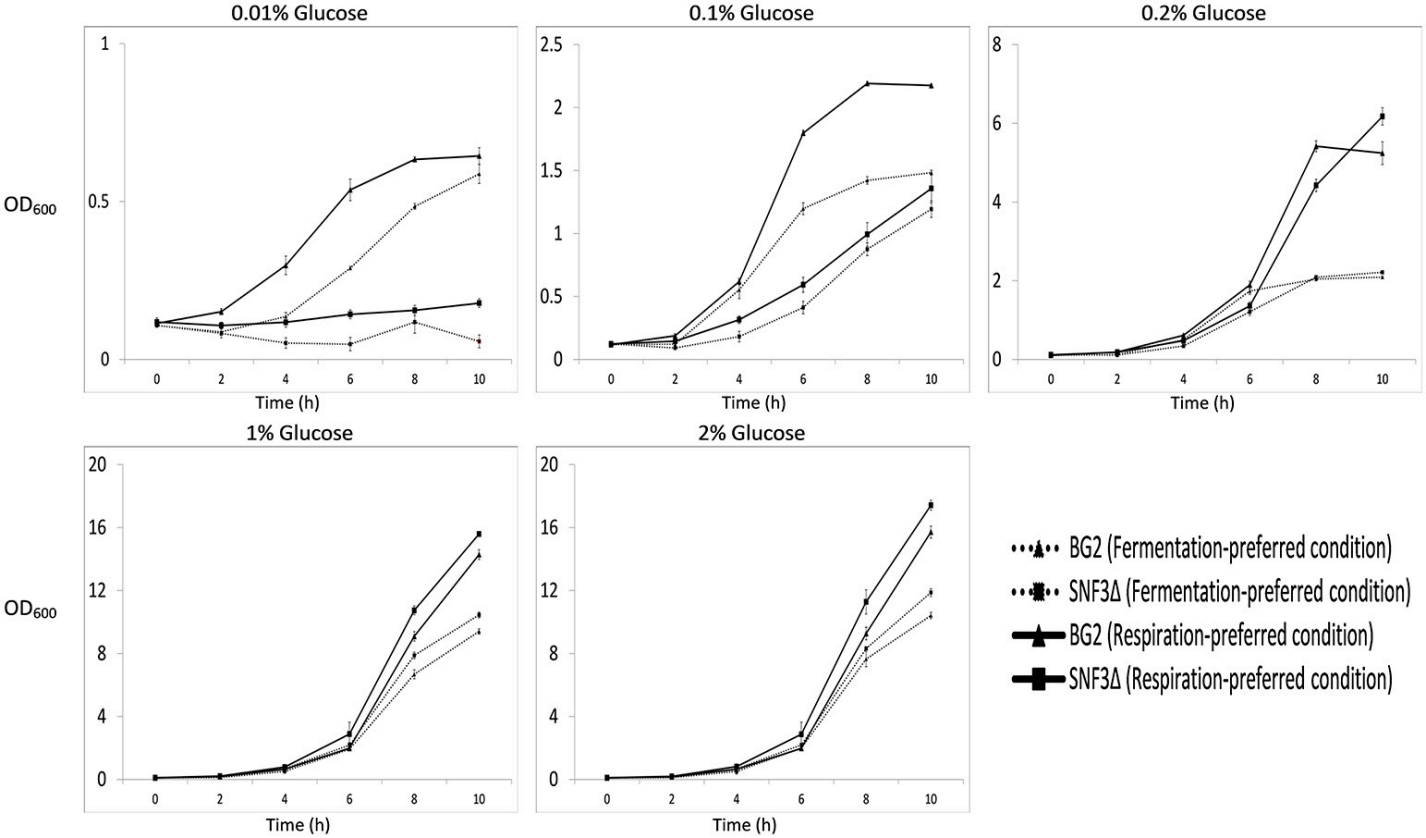
**Growth profile of ***Candida glabrata*** BG2 and ***SNF3***Δ in five difference glucose concentrations tested for both fermentation and respiration-preferred condition**.

**Figure 2:**
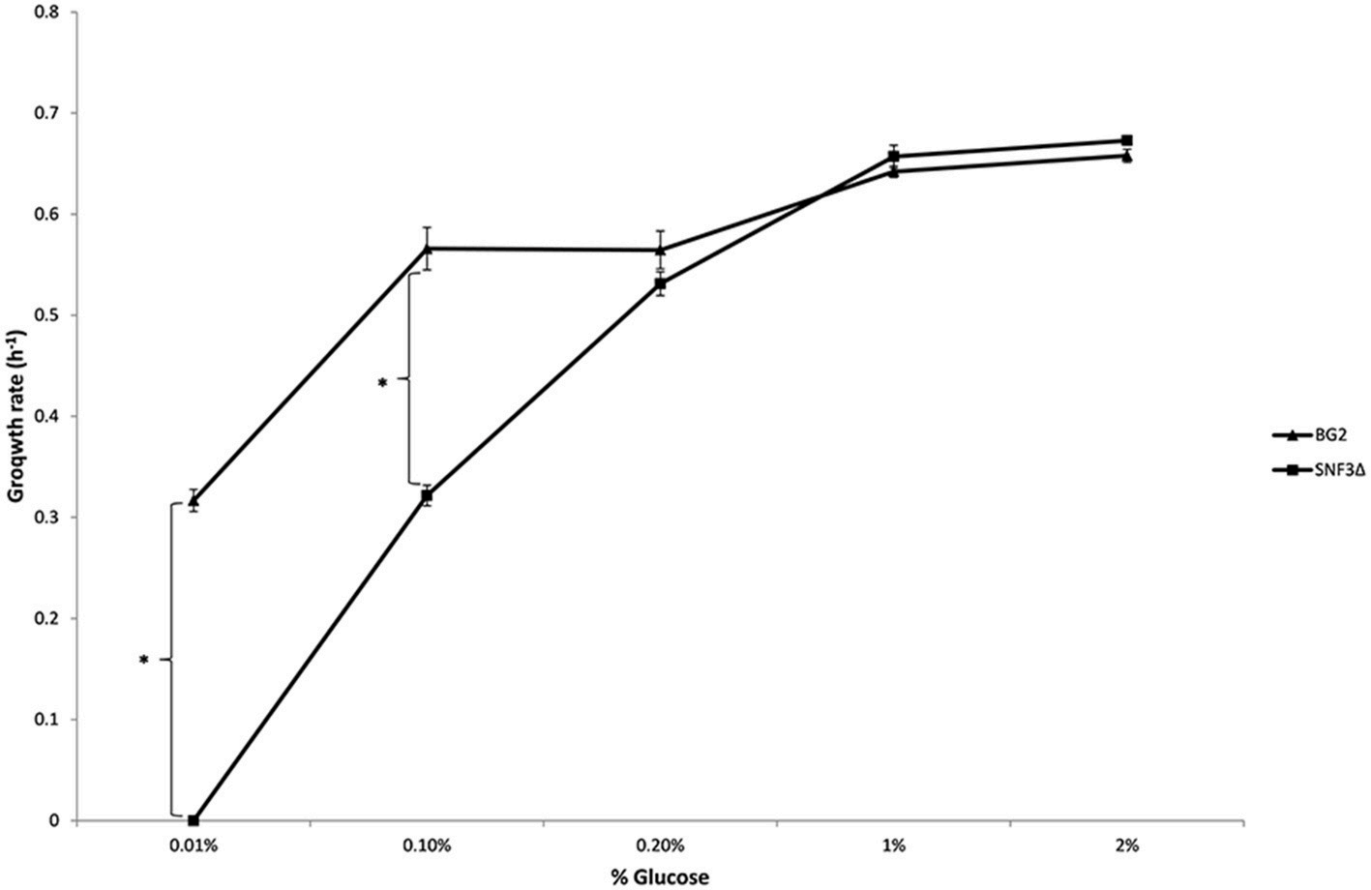
**Growth rate of ***Candida glabrata*** BG2 and ***SNF3***Δ in five differences glucose concentrations with respiration-preferred condition**. Significant differences (indicated by ^*^) were found between wild type and mutant under low glucose environments: 0.01 and 0.1% (*p*-value < 0.05).

**Figure 3:**
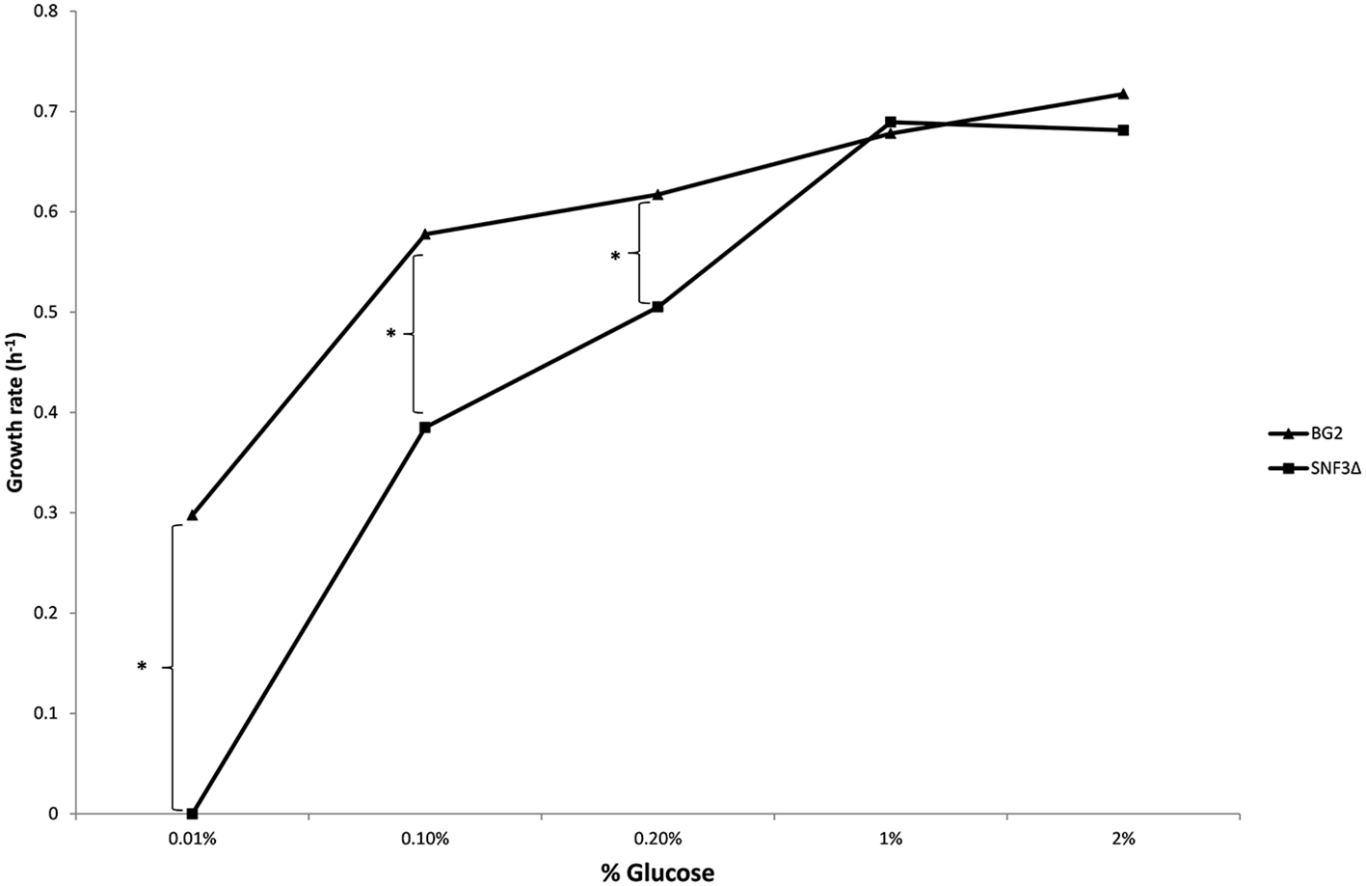
**Growth rate of ***Candida glabrata*** BG2 and ***SNF3***Δ in five differences glucose concentrations with fermentation-preferred condition**. Significant differences (indicated by ^*^) were found between wild type and mutant under low glucose environments: 0.01, 0.1, and 0.2% (*p*-value < 0.05).

The inabilities of *SNF3*Δ to grow in low glucose environments were demonstrated in both shaking and static condition (Figures 1–3). After 10 h of incubation, the growth rate of *SNF3*Δ strain was significantly reduced (*p*-value < 0.05) in 0.01 and 0.1% glucose environment for respiration preferred-condition (shaking) and in 0.01, 0.1, and 0.2% glucose environment for fermentation preferred-condition (static). However, deletion of *SNF3* did not weaken the growth of *SNF3*Δ strain in higher glucose environment (1 and 2%; Figures 1–3). These observations highlighted the role of *SNF3* in sustaining the growth of *C. glabrata*, particularly in low glucose for both respiration and fermentation-preferred environment. Furthermore, *SNF3* is deemed to be more important in fermentation process where the growth defect of *SNF3*Δ was found to be more severe (extended up to 0.2% glucose) in fermentation-preferred condition. In respect of the data obtained, which suggested the deleterious effect of *SNF3*Δ is seen only in low glucose environment, the subsequent assays including biofilm formation and amphotericin B susceptibility assays were carried out in glucose limited environment (0.01 and 0.1%).

### Deletion of *SNF3* gives no effect in the biofilm formation capability of *C. glabrata* in glucose-limited environments

**Figure 4:**
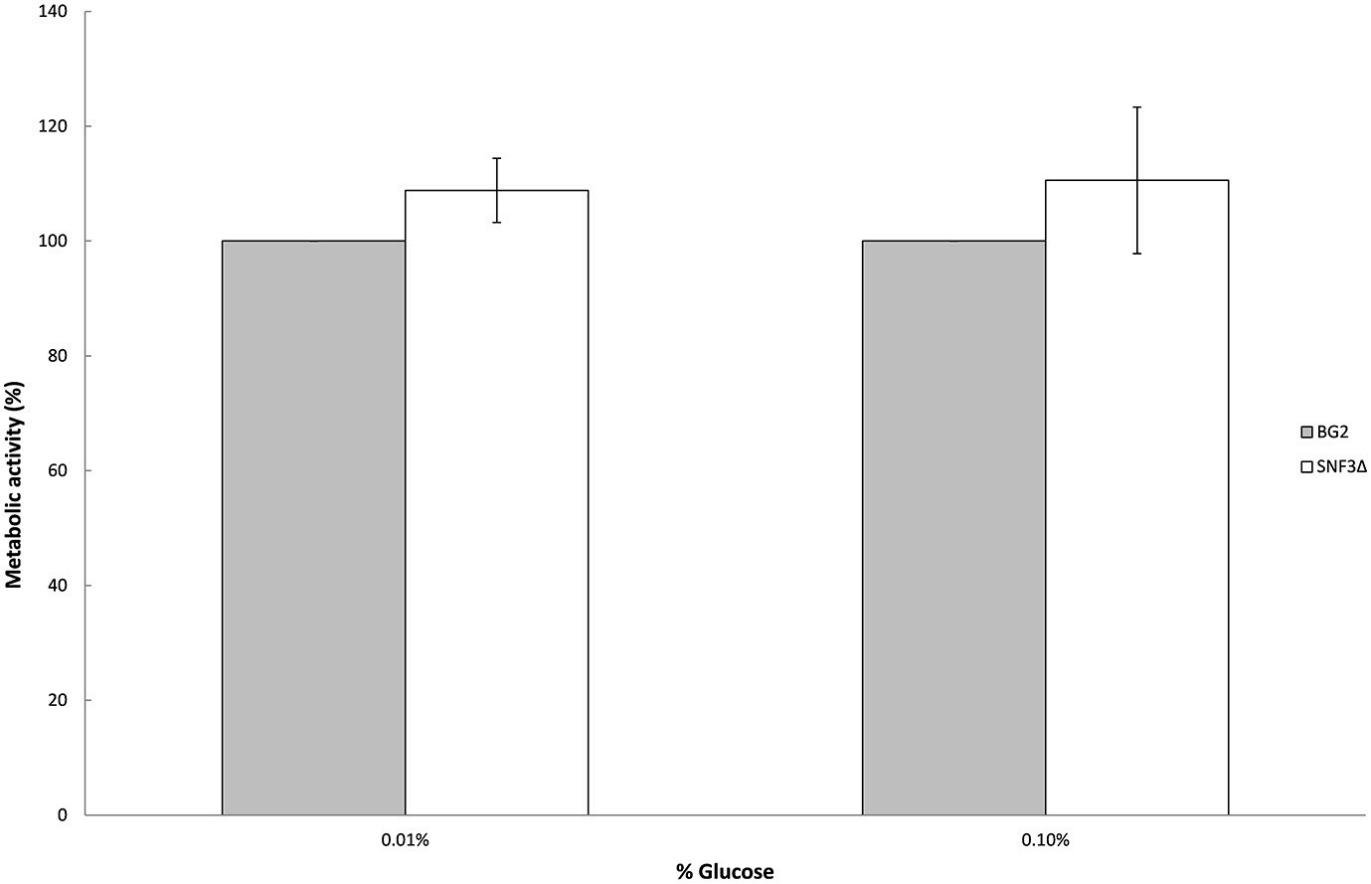
**Biofilm formation activity of ***Candida glabrata*** BG2 and ***SNF3***Δ strains under 0.01 and 0.1% glucose concentration**. Unpaired *T*-test was carried out for the statistical analysis to examine the significant difference between BG2 and *SNF3*Δ and no significant difference was found.

Previous study demonstrated the effects of glucose levels in directing *C. albicans* to form biofilm. *Candida albicans* tends to form biofilm in low glucose environment and lives in planktonic form in higher glucose environment (Uppuluri et al., 2010; Ng et al., 2015b). The sensitivity of *SNF3* in responding to surrounding glucose leads to the thought whether this putative high affinity glucose sensor could contribute in detecting the flow of surrounding glucose and therefore orchestrates the biofilm/planktonic living form of *Candida* species in accordance to the availability of glucose. Result showed *SNF3* did not participate in the biofilm formation of *C. glabrata* in low glucose environment as no significant differences were found between BG2 and *SNF3*Δ in the 0.01 and 0.1% glucose tested, respectively (Figure 4).

### The loss of *SNF3* makes *C. glabrata* more vulnerable to amphotericin B treatment in low glucose concentration environment

**Figure 5:**
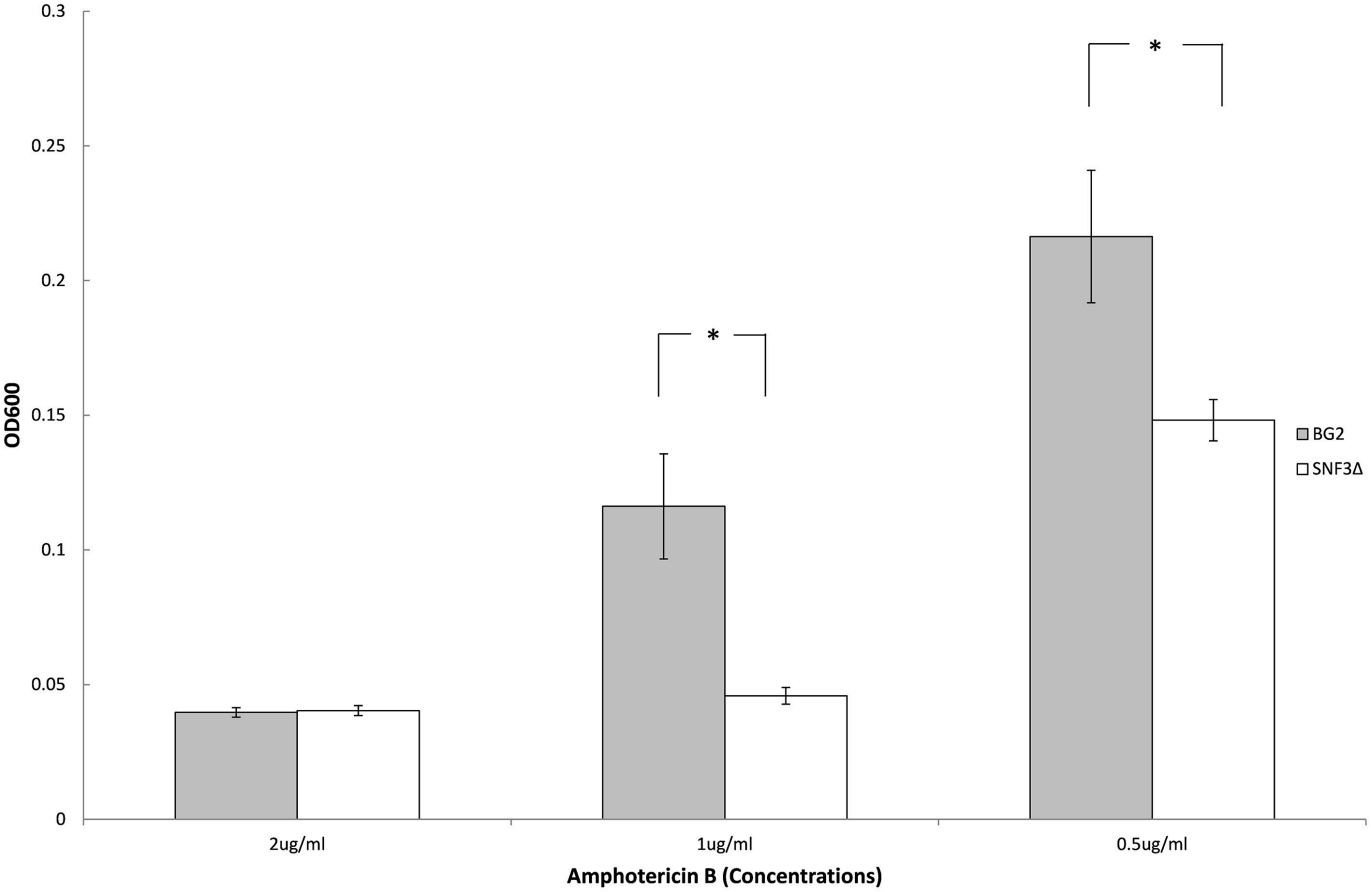
**Survivability of ***Candida glabrata*** BG2 and ***SNF3***Δ strains under treatment of three different concentrations of amphotericin B in 0.1% glucose**. Unpaired *T*-test was carried out for the statistical analysis to examine the significant differences (indicated by ^*^) between BG2 and *SNF3*Δ (*p*-value < 0.01).

Previous study demonstrated the ability of *Candida* species to withstand antifungal is affected by the type of carbon sources and levels (Ene et al., 2012; Mota et al., 2015; Ng et al., 2015b). With the aim to elucidate further the possible role of *SNF3* in regulating the fitness of *C. glabrata*, the ability of both strains to withstand amphotericin B in low glucose environment was tested. However, the complete retarded growth of *SNF3*Δ in 0.01% glucose leads to the inability in the effort to set up an unstressed control for the calculation of survival percentage. Thus, only 0.1% glucose was tested in this assay. The growth of both wild type and *SNF3*Δ strain were arrested at 2 μg/mL of amphotericin B. The wild type strain was able to resist amphotericin B at 1 μg/mL while complete inhibition was observed in the *SNF3*Δ strain (*p*-value < 0.01). The wild type showed a better growth in comparison to *SNF3*Δ strain at 0.5 μg/mL amphotericin B (Figure 5). These data established the fact that glucose sensing by the *SNF3* gene may contribute to the ability of *C. glabrata* in withstanding the effects of amphotericin B under low glucose environment.

### *SNF3*Δ strain shows reduced growth in macrophages

**Figure 6:**
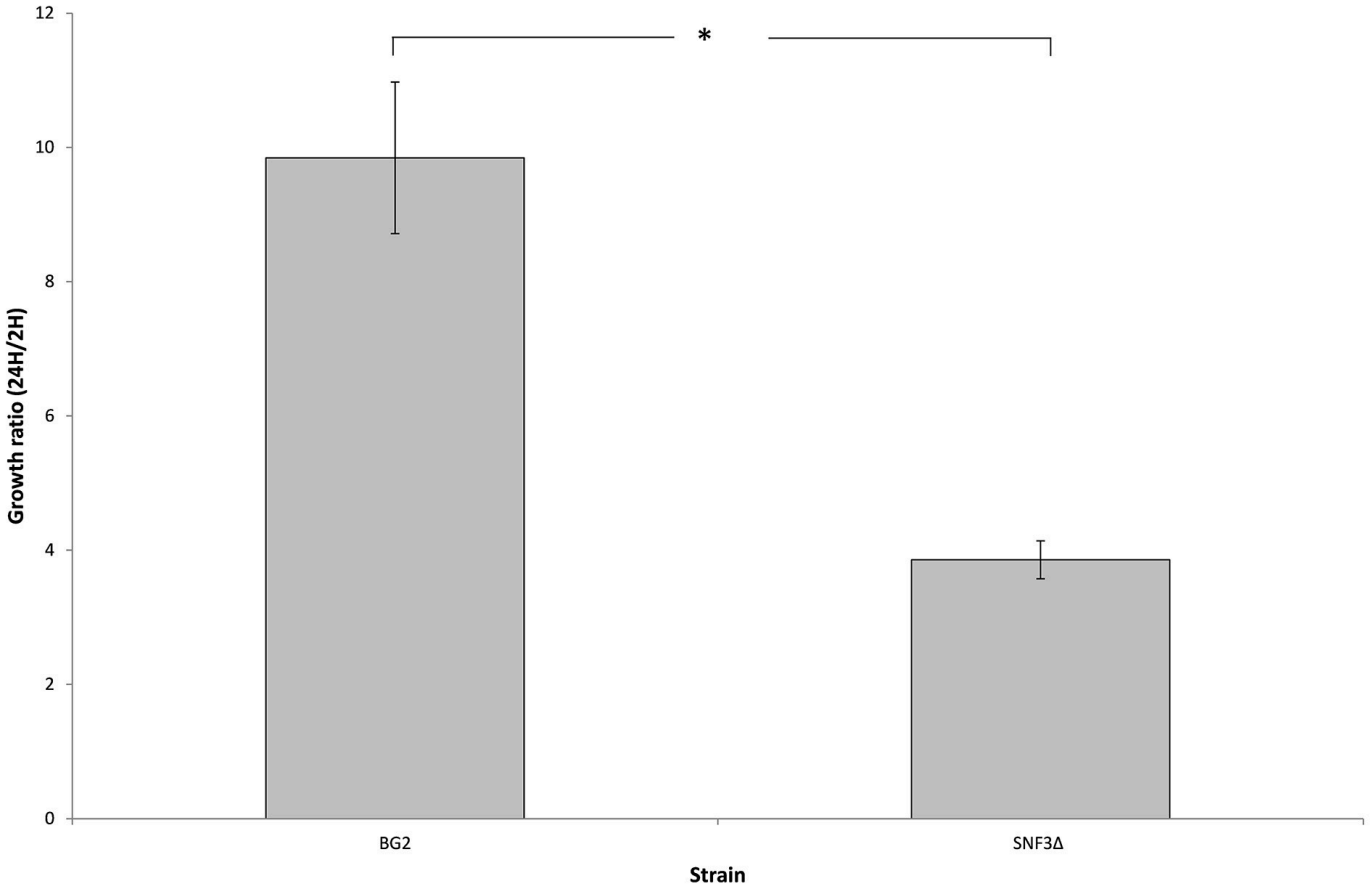
**The survival ratio of ***Candida glabrata*** BG2 and ***SNF3***Δ strains recovered from macrophages at 24 h vs. 2 h after co-cultivation**. Unpaired *T*-test was carried out for the statistical analysis to examine the significant differences (indicated by ^*^) between BG2 and *SNF3*Δ (*p*-value < 0.01).

The microenvironment in macrophage is always linked to nutrient-limited environment, particularly in glucose availability. In order to validate the possible role of *SNF3* in promoting the fitness of *C. glabrata* under glucose-limited environment, the survivability of macrophage trapped- *C. glabrata* was assayed in an *ex vivo* manner. Results demonstrated a significant reduced growth (*p*-value < 0.01) of the internalized mutant strain in comparison to wild type strain (Figure 6) and this suggests the essential role of *SNF3* in supporting the survivability of *C. glabrata* upon macrophage engulfment.

### Deletion of *SNF3* affects the expression of downstream hexose transporters *(HXTs)*

**Figure 7:**
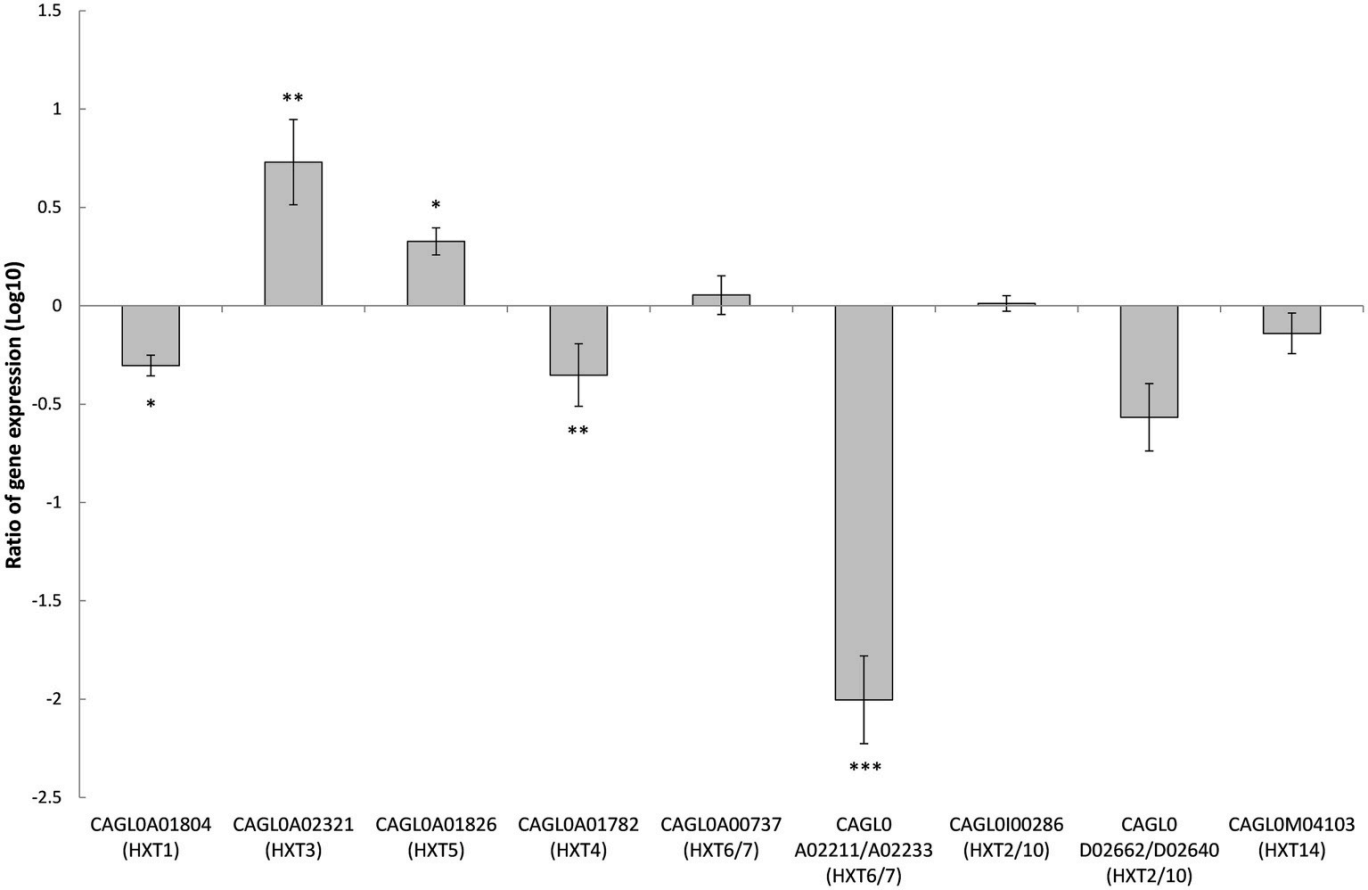
**Comparison of expression ratios (Log_10_) for the ***Candida glabrata*** hexose transporters (***HXT***s) after the knockout of ***SNF3*****. ^*^p < 0.1, ^**^p < 0.05, ^***^p < 0.01.

**Figure 8:**
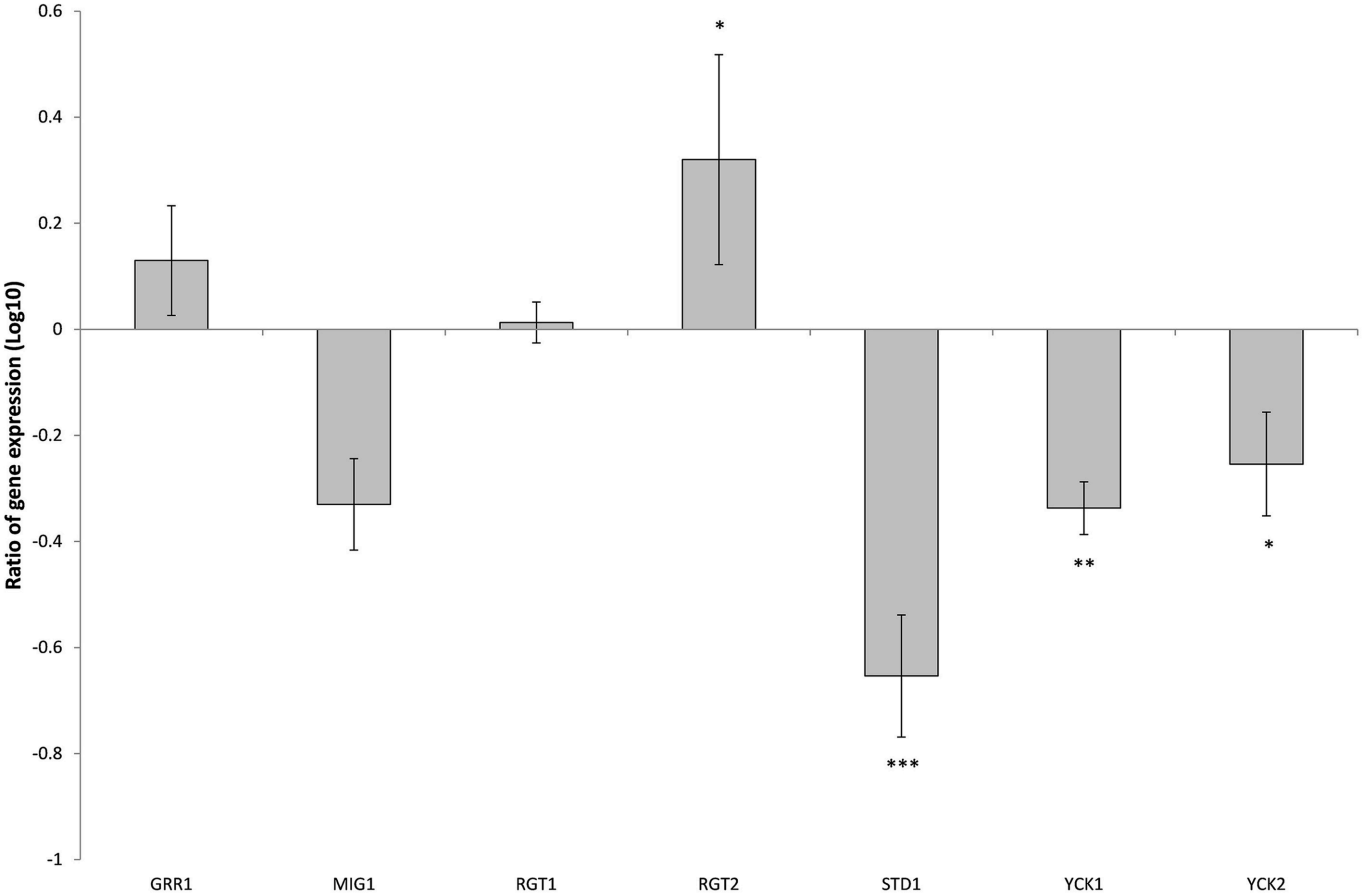
**Comparison of expression ratios (Log_10_) for the ***Candida glabrata*** Sugar Receptor Repressor (SRR) related genes after the knockout of ***SNF3*****. ^*^p < 0.1, ^**^p < 0.05, ^***^p < 0.01.

There are 11 hexose transporters found in *C. glabrata*. The expressions of these hexose transporters were examined and compared between wild type and *SNF3*Δ strain. Out of 11 hexose transporters, only nine hexose transporters were studied because the nucleotide sequences of putative hexose transporters CAGL0A02211 and CAGL0A02233 were found to be 96% similar while CAGL0A02662 and CAGL0A02640 displayed 100% similarity. The high similarity among these hexose transporters caused the inability in primer design for the expression study of those genes. Out of nine hexose transporters, six of them were affected with the deletion of *SNF3*, where four of them (CAGL0A1804_*HXT1*, CAGL0A01782_*HXT4*, CAGL0A02211/2233_*HXT6/7*, and CAGL0D02662/2640_*HXT2/10*) were down regulated while two (CAGL0A02321_*HXT3* and CAGL0A01826_*HXT5*) were up regulated (Figure 7). In addition, deletion of *SNF3* resulted in down-regulation of *STD1, YCK1*, and *YCK2*, which serve as the downstream messengers of *SNF3* to modulate the expression of hexose transporters (Figure 8). Nevertheless, the expression of *RGT2* was up regulated while expression of *RGT1, GRR1*, and *MIG1* did not change significantly with the deletion of *SNF3* (Figure 8). These observations suggest the significant role of *SNF3* in regulating the signaling pathway of glucose uptake mechanism.

## Discussion

Data presented in present study is suggestive of the role of *SNF3* as high affinity glucose sensor in *C. glabrata*, which is essential for it to grow in glucose-limited environment. *SNF3* appeared to be important for the growth of *C. glabrata* in both respiration and fermentation preferred condition with low glucose environments (0.01 and 0.1%) and up to 0.2% glucose in fermentation preferred condition. Brown et al. (2006) demonstrated the deletion of glucose sensor, *HGT4* in *C. albicans* attenuates its ability to grow only in the fermentation-preferred condition and low level of fermentable carbon source (0.2%). Data suggest high affinity glucose sensor appears to be more essential in *C. glabrata* than in *C. albicans*. The dissimilarities observed in both the *Candida* species could be due to the differences in their nature of glucose utilization. *C. glabrata* is identified as Crabtree-positive yeast or aerobic fermenter where, it prefers fermentation over respiration and produces ethanol even there is presence of oxygen (Van Urk et al., 1990), while *C. albicans* is known as Crabtree-negative yeast or respiratory yeast, which prefers respiration whenever there is presence of oxygen. The preferred-fermentation in Crabtree-positive yeast produces only two ATP per glucose, in comparison to 36/38 ATP per glucose produced in respiration. Owing to the preferred-inefficient mode of metabolism, Crabtree-positive yeast is found to exhibit higher glucose consumption rate than Crabtree-negative yeast (Van Urk et al., 1990; Fleck et al., 2011). Therefore, the presence of two specialized glucose sensors in *C. glabrata* (Palma et al., 2009; Ng et al., 2015a) may contribute to the higher glucose uptake sensitivity in order to fulfill its ATP demands and removal of this high affinity glucose sensor lead to detrimental effect on the growth of *C. glabrata* in low glucose environment (Figures 1–3). Unlike *C. glabrata*, there is only one high affinity glucose sensor (*HGT4*) found in *C. albicans* (Brown et al., 2006). The capability of *C. albicans* to assimilate both fermentable and non-fermentable carbon sources at the same time suggests *C. albicans* has evolved distinctively to adapt itself by not relying solely on glucose for its growth in hostile host niche with limited glucose availability (Sandai et al., 2012). Thus, a single glucose sensor is probably sufficient to support the life process of *C. albicans*. However, it is still unclear whether *C. glabrata* is equipped with the same metabolic flexibility. The presence of the two glucose sensors with different affinity in *C. glabrata* similar to the ones found in *S. cerevisiae* suggests it would behave more like *S. cerevisiae*. The baker's yeast is unable to assimilate both fermentable and non-fermentable carbon sources at the same time (Sandai et al., 2012). Further investigation on the carbon metabolic flexibility in *C. glabrata* is warranted as this could provide insight into the metabolic adaptation on the disease progression of this fungal pathogen.

Apart from glucose sensing and uptake mechanism, the expression of glucose sensor gene *HGT4* in *C. albicans* is deemed to be regulated by macrophage engulfment, antifungal mechanism, and biofilm formation activity of yeast (Barker et al., 2004; Liu et al., 2005; Brown et al., 2006). The deletion of *SNF3* indeed diminished the capability of *C. glabrata* to withstand the macrophage challenge and amphotericin B treatment but did not affect its biofilm formation activity. Data presented demonstrated the importance of *SNF3* in supporting the growth of *C. glabrata* under low glucose environment in the growth profiling assay. This observation was extended further to the nutrient-limiting microenvironment of macrophage. Macrophage is critically important in building up an immunological barrier to counter infectious agents through its unique nutritional seal off and oxidative stress to destroy engulfed intruders (Kaur et al., 2006). Previous study demonstrated the capability of *C. glabrata* to perform autophagy for the nutritional scavenging and recycling in order to sustain its growth upon phagocytosis (Roetzer et al., 2010). Data (Figure 6) suggests in addition to autophagy mechanism, glucose sourcing and uptake are also important in aiding *C. glabrata* to sustain prolonged phagocytosis. The absence of *SNF3* may result in the inability of *C. glabrata* to absorb sufficient glucose to perform basic physiological function or even to initiate autophagy mechanism and therefore lead to the diminished growth. On the other hand, deletion of *SNF3* did not affect the capability of *C. glabrata* to form biofilm under low glucose condition as expected. Data presented suggest there is probably another sensor in *C. glabrata* but not *SNF3* that assists in detecting the nutrient flow in environment. Further investigation is warranted for a clearer picture on how this pathogenic yeast senses and alters its lifestyle to adapt itself in such environment where abrupt change of nutrients takes place.

**Figure 9:**
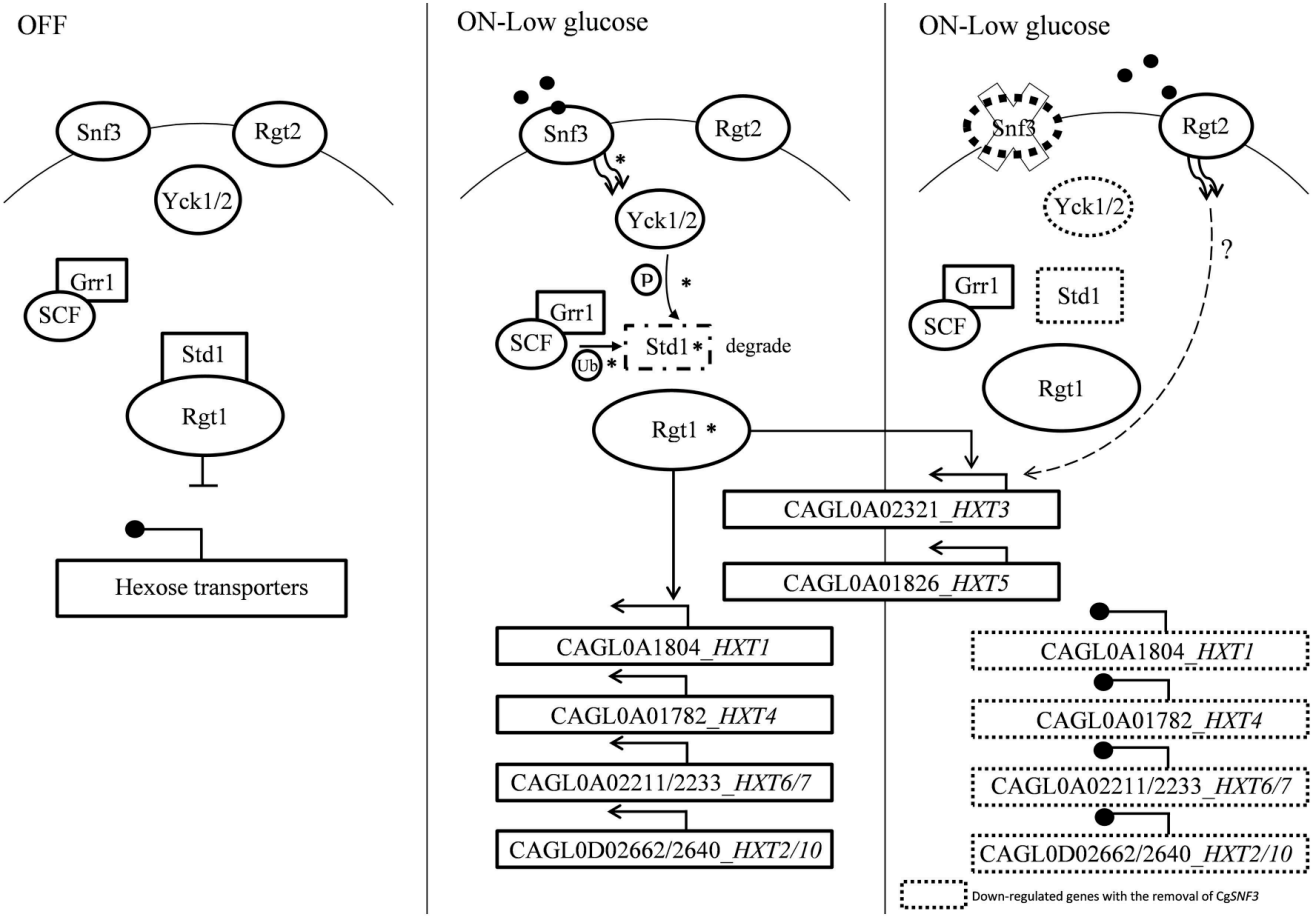
**A model of glucose sensing in ***Candida glabrata*** under low glucose environment**. The part of the pathway labeled with asteisks inferred from published works done on *S. cerevisiae* (Rolland et al., 2002; Santangelo, 2006; Gancedo, 2008). Hexose transporters are repressed by Std1-bounded-Rgt1 when there is no stimulation from glucose sensor located in the cell membrane. Presence of low concentration of glucose induced signal from high affinity glucose sensor, Snf3 to the phosphorylation of Std1 by the Yck kinase. Phosphorylated Std1 is then subjected to the SCF^Grr1^—mediated ubiquitination and degraded by proteasome. Degraded Std1 results in the activation of Rgt1, which then leads to derepression of downstream hexose transporters. Deletion of *SNF3* gives rise to the disruption of hexose transporters expression and glucose uptake mechanism, therefore leads to the interference of *Candida glabrata* fitness under low glucose environment. However, the possible interaction between *RGT2* and downstream *HXT3*/*HXT5* (labeled with dotted line) is remains unclear and requires further investigation.

The transcriptional analysis on selected hexose transporters (*HXT*s) revealed that almost half (four out of nine) hexose transporters were down regulated with the removal of *SNF3*, together with the down-regulation of downstream casein kinase (*YCK1* and *YCK2*) and *STD1* (Figures 7, 8). The disruption of the signaling pathway for high affinity hexose transporters explained the compromised fitness of *C. glabrata* under low glucose environment (Figures 1–3) as this triggers the failure in transporting sufficient glucose to support its growth. In addition, data presented concurs with the view that the expression of transcription regulator, *RGT1* is regulated by the glucose concentration but not affected by the signal generated from glucose sensors (Özcan and Johnston, 1999) as the expression of *RGT1* remain unchanged even with the missing signal from *SNF3*. However, the direct regulation of glucose concentration on the expression level of *RGT1* is still not fully understood. Nonetheless, the shutting down of these four hexose transporters did not diminish the growth of *C. glabrata* completely as there are two other hexose transporters that were still actively expressed namely the CAGL0A0232 (*HXT3*) and CAGL0A01826 (*HXT5*), together with the up regulation of *RGT2*. This could be a compensatory mechanism used by *C. glabrata* to compensate the loss of *SNF3* with the activation of *RGT2*. Notably, these *HXT3* and *HXT5* were regarded as key hexose transporters for *C. glabrata* in low glucose environment from our previous work (Ng et al., 2015a). Nevertheless, this compensatory mechanism still failed to salvage *C. glabrata* from glucose uptake crisis as the growth defect is still significant (*p*-value < 0.05; Figures 1–3) in the absence of *SNF3*. We opine the compensation of glucose uptake by *HXT3* and *HXT5* is insufficient to provide the amount of glucose needed and this highlights the importance of four other repressed *HXTs* in supporting the growth of *C. glabrata* under low glucose environment. In addition, the capability of *RGT2* to induce expression of *HXT3* and *HXT5* supports the view that *SNF3* and *RGT2* have separate but overlapping functions. Özcan et al. (1998) demonstrated the capability of *SNF3* in *S. cerevisiae* to restore the expression of *HXT1* (supposedly induce by *RGT2*) by 64%, in a *RGT2* mutant. This observation suggests a complex and interconnected regulatory pathway of glucose sensing and uptake mechanism in yeast. From the data obtained, a model of glucose sensing in *C. glabrata* through the modulation of *SNF3* is illustrated based on the understanding of the homolog and the inferred glucose sensing mechanism in *S. cerevisiae* (Figure 9). Further work is warranted, as the compensatory mechanism proposed here is still not fully deciphered. In addition, effort to study the transcriptional profile of the highly homologous *HXT*s genes using other approach should be carried out. With more complete information on the role of each hexose transporters present in *C. glabrata*, a clearer and more comprehensive picture on the role of *SNF3* in SRR pathway will be achieved.

In conclusion, our results thus far suggest the important role of *SNF3* in *C. glabrata* in the expression of hexose transporters under low glucose environment. We also highlight the vital role of *SNF3* in promoting *C. glabrata* growth, resistant toward amphotericin B under glucose limited environment and macrophage engulfment by governing the glucose uptake mechanism. These results suggest *SNF3* could be a potential factor for *C. glabrata* to survive and thrive in host niches with limited glucose availability. Further investigation such as RNA-sequencing and comparative proteomic study could be carried out for the analysis of global transcriptomes and validation of the obtained result. Owing to the essential role of glucose on metabolic network of organism, further exploration on the glucose sensing mechanism highlighted in current study could contribute in the discovery of novel drug target and help in controlling the emergence of *C. glabrata*.

## Author contributions

TS, LT, and DS designed the experiments. TS, SY, and PR carried out the experiments. TS analyzed and interpreted the data. TS and LT wrote the manuscript with critical revision for important intellectual content from MD, DS, and PP.

### Conflict of interest statement

The authors declare that the research was conducted in the absence of any commercial or financial relationships that could be construed as a potential conflict of interest.
